# Early intensive hand rehabilitation after spinal cord injury ("Hands On"): a protocol for a randomised controlled trial

**DOI:** 10.1186/1745-6215-12-14

**Published:** 2011-01-17

**Authors:** Lisa A Harvey, Sarah A Dunlop, Leonid Churilov, Ya-Seng Arthur Hsueh, Mary P Galea

**Affiliations:** 1 Rehabilitation Studies Unit, Northern Clinical School, Sydney School of Medicine, University of Sydney, - PO Box 6, Ryde, NSW, 1680, Australia; 2 School of Animal Biology (M317), The University of Western Australia, Crawley, Western Australia, 6009, Australia; 3 Florey Neuroscience Institutes (National Stroke Research Institute) and Department of Mathematics and Statistics, The University of Melbourne, Parkville, Victoria, 3010, Australia; 4 Centre for Health Policy, Programs and Economics, School of Population Health, The University of Melbourne; 5 Physiotherapy, The University of Melbourne, Parkville, Victoria, 3010, Australia

## Abstract

**Background:**

Loss of hand function is one of the most devastating consequences of spinal cord injury. Intensive hand training provided on an instrumented exercise workstation in conjunction with functional electrical stimulation may enhance neural recovery and hand function. The aim of this trial is to compare usual care with an 8-week program of intensive hand training and functional electrical stimulation.

**Methods/design:**

A multicentre randomised controlled trial will be undertaken. Seventy-eight participants with recent tetraplegia (C2 to T1 motor complete or incomplete) undergoing inpatient rehabilitation will be recruited from seven spinal cord injury units in Australia and New Zealand and will be randomised to a control or experimental group. Control participants will receive usual care. Experimental participants will receive usual care and an 8-week program of intensive unilateral hand training using an instrumented exercise workstation and functional electrical stimulation. Participants will drive the functional electrical stimulation of their target hands via a behind-the-ear bluetooth device, which is sensitive to tooth clicks. The bluetooth device will enable the use of various manipulanda to practice functional activities embedded within computer-based games and activities. Training will be provided for one hour, 5 days per week, during the 8-week intervention period. The primary outcome is the Action Research Arm Test. Secondary outcomes include measurements of strength, sensation, function, quality of life and cost effectiveness. All outcomes will be taken at baseline, 8 weeks, 6 months and 12 months by assessors blinded to group allocation. Recruitment commenced in December 2009.

**Discussion:**

The results of this trial will determine the effectiveness of an 8-week program of intensive hand training with functional electrical stimulation.

**Trial registration:**

NCT01086930 (12^th ^March 2010)

ACTRN12609000695202 (12^th ^August 2009)

## Background

The incidence of spinal cord injury (SCI) varies between countries but is estimated at 10 to 83 per million, per year with most injured under the age of 25 years [1]. More than one third of these individuals sustain an injury that causes damage to the spinal cord in the cervical region and results in tetraplegia [1]. Most people with tetraplegia remain wheelchair-dependent and reliant on others for physical care. Importantly, however, limited hand and upper limb function is often more disabling and of greater importance to them than their inability to walk [2,3]. Even modest improvements in hand function can have life-changing implications. For example, a small amount of finger movement enables a person with tetraplegia to use a keyboard, press a switch, scratch the face and turn the page of a book. The ability to do these simple tasks reduces dependency on others, improves potential for employment and enhances quality of life.

There is evidence to suggest that intensive task-specific training can enhance hand function in people with tetraplegia [4,5]. Intensive training with superimposed functional electrical stimulation (FES) may be particularly therapeutic especially in individuals with poor grasp [6-8]. It is believed that this combination of therapies provides the damaged spinal cord with excitation from the sensorimotor cortex along with intensive sensory input from the periphery. Neural bombardment of this kind on the damaged spinal cord may promote neural plasticity and, in particular, may provide the critical stimulus required to elicit neurophysiologic and structural re-organisation of the relevant pathways [9].

**Figure 1:**
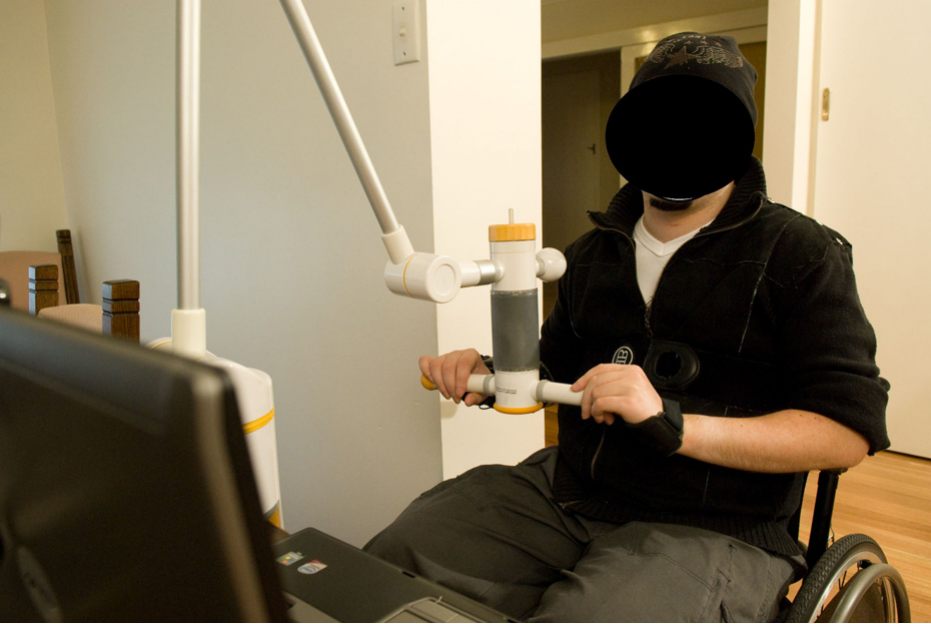
**The instrumented exercise workstation (ReJoyce) demonstrating the different manipulanda**.

Recent advances in computer-game technology provide innovative ways of encouraging patients to engage in intensive task-specific training [10]. Here we describe the protocol for a randomised controlled trial using FES and an exercise workstation (ReJoyce, Rehabtronics Inc. Edmonton, Canada). Patients wear a behind-the-ear blue tooth sensor that is triggered by tooth clicks and sends radio signals to a customised muscle-stimulator garment worn on the forearm. This system enables people with tetraplegia to independently stimulate their own hands to open or close, allowing grasp and release of a variety of manipulanda (pinch, squeeze, grasp, twist, lift, push, pull; see Figure 1). The patients thus use their hands to control the manipulanda and play computer games while practising different hand grasps. The games are graded according to hand function so even patients with severe upper limb paralysis can participate and, similarly, patients can play progressively more difficult games as they improve. The technology thus provides a way of encouraging patients to do large numbers of different hand movements within a dynamic environment. The primary aim of this trial therefore is to determine the effect on hand function of an intensive task-specific hand training program provided with FES through an instrumented exercise workstation. The secondary aim is to determine possible benefits on muscle strength, sensation, function, and quality of life.

## Methods/Design

### Funding

The trial is funded by theVictorian Neurotrauma Initiative, NSW Lifetime Care and Support Authority, The University of Melbourne and The University of Western Australia.

### Design

A multi-centre randomised controlled trial will be undertaken. One hand of each participant will be identified as the target hand. The target hand of participants will be randomised to the experimental or control group. Control participants will receive usual care while experimental participants will receive usual care plus an intensive 8-week program directed at the target hand. The trial will be conducted through seven SCI units in Australia and New Zealand. Ethical approval has been obtained from the Human Research Ethics Committee at each site and the University of Melbourne (HREC 0932764.1). Participants will be provided with information sheets and written informed consent will be obtained prior to recruitment and baseline assessment. Subject recruitment commenced December 2009 and will finish July 2011.

### Participants

**Figure 2:**
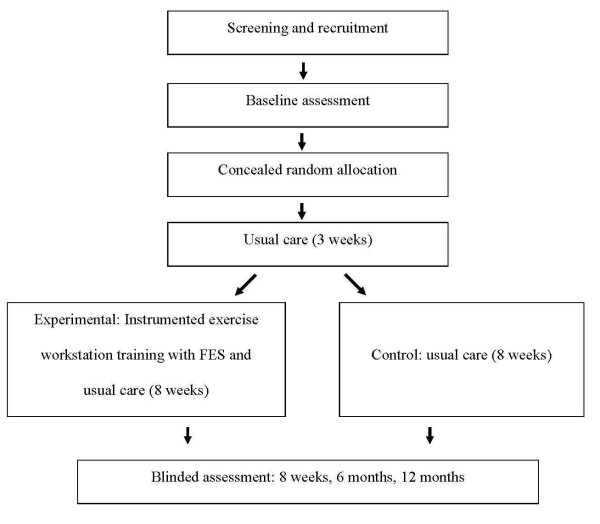
**Flow diagram of the trial**.

Seventy-eight participants with recently-acquired tetraplegia undergoing inpatient rehabilitation in one of the seven participating SCI units will be recruited from a consecutive sample of admissions (see Figure 2). Therapists at each SCI unit will screen participants. One hand of each participant will be identified as the target hand according to the criteria below. In situations where both hands meet the inclusion criteria, the hand deemed most likely to benefit from intensive training by the treating therapist will be selected.

### Inclusion criteria

Participants will be included if they:

1. have sustained a SCI within the preceding 6 months

2. are currently receiving inpatient rehabilitation through one of the seven participating SCI units

3. will remain in the SCI unit for 12 weeks as part of standard rehabilitation

4. are 16 years of age or older and able to provide informed consent

5. have a motor complete or incomplete SCI at the neurological level of C2 to T1 (as per the International Standards of Neurological Classification for SCI)

6. have reduced ability to grasp with the target hand

7. are able to tolerate sufficient FES to enable the target hand to grasp and release

8. have the potential to benefit from the experimental intervention according to the judgment of the treating therapist

### Exclusion criteria

Participants will be excluded if they:

1. have any other type of neurological injury affecting the target hand (e.g. brachial plexus or peripheral nerve injuries)

2. have had trauma or surgery to the target hand or upper limb within the last 12 months

3. have had amputation of any digits on the target hand

4. are not able to sit out of bed for at least two hours per day over three consecutive days

5. have extensive fixed contractures in the upper limb of the target hand preventing use of the instrumented exercise workstation

6. have severe spasticity in the target hand or upper limb preventing use of the instrumented exercise workstation

7. are unable to attend the 6 month and 12 month follow-up assessments

8. are likely to undergo hand surgery in the target hand in the next year

9. are likely to experience autonomic dysreflexia or hypotension in response to FES

10. have any contraindications to FES such as cardiac pacemaker, epilepsy, forearm fracture or pregnancy

11. have intracranial metal implants

12. have impaired vision or are unable to view a computer screen

13. have any other serious medical condition likely to influence cooperation and adherence to the protocol including malignancies and psychiatric, behavioural or drug-dependency problems

### Randomisation

Participants will be randomly assigned to either control or experimental group with a 1:1 allocation as per a computer generated randomisation schedule stratified by site and the baseline score of the Action Arm Research Test (ARAT; <= 21 versus >21) using permuted blocks of random sizes. The block sizes will not be disclosed, to ensure concealment.

Prior to commencement of the trial, an independent researcher with no clinical involvement in the trial will use a computer random number generator to produce the randomisation schedule. Randomisation will occur after completion of baseline assessments by contacting an administrator independent of the recruitment process and located off site at Neuroscience Trials Australia, for allocation assignment. A participant will be considered to have entered the trial once his/her randomisation is revealed.

### Intervention

#### Control participants

Control participants will receive usual care and will not receive any electrical stimulation to the target hand or upper limb nor will they be exposed to the instrumented exercise workstation.

#### Experimental participants

In addition to the usual care provided to all participants, experimental participants will receive one hour of one-to-one hand training directed at the target hand with a research therapist, five times per week for 8 weeks. The training will consist of an intensive task-specific hand training program provided through an instrumented exercise workstation (ReJoyce) in conjunction with FES. The hand activities will involve playing computer games while practising functional tasks (including reaching, grasping, manipulating, pulling, rotating and releasing) using different manipulanda (see Figure 1).

The exercises and computer games will be progressed so that, as hand function improves, more difficult hand exercises and games will be introduced. Each training session will be one hour long and participants will be required to use the instrumented exercise workstation and FES as much as possible during this time.

The FES will be provided through 5 cm diameter electrodes embedded in wetted cloth pads backed with stainless steel mesh. The electrodes will be incorporated into a customised garment. The cathode of each electrode pair will be placed over the proximal end of each target muscle. The reference electrode will be placed on the dorsal surface of the forearm and just proximal to the wrist joint. The FES will be provided in trains of stimuli (50 per second: 200 μs biphasic, current-controlled pulses). The FES will be administered to any or all of the muscles that facilitate opening or closing of the hand including the flexors and extensors of the wrist, fingers and thumb.

FES will be triggered by participants clicking their teeth. The tooth clicks (vibrations) will be detected by a wireless earpiece, similar to a hearing aid, which sends a radio signal to the stimulator garment. This in turn stimulates the hand to open or close, allowing participants to grasp or release objects. The stimulator system has been tested and approved by the Canadian Standards Association. As it is not yet approved in Australia, the stimulator is being used for this trial under the Clinical Trials Notification Scheme of the Therapeutic Goods Administration of Australia.

If participants miss any treatments during the 8-week intervention period, the missed sessions will be offered to participants on weekends or during an optional additional week at the end of the 8-week intervention period.

#### All participants

All participants will continue to receive usual care for the hand and upper limb as typically provided by their SCI units. Usual care will be individualised to the needs of participants but will involve at least three 15-minute sessions per week of one-to-one therapy specifically directed at the target hand. This may consist of practising any or all of the following functional activities: moving checkers, grasping and releasing objects, manipulating objects, turning keys, pouring water and opening jars. In addition, all participants will receive any of the following as typically provided by participants' usual treating therapists as part of regular physiotherapy as well as vocational, recreational and occupational therapy:

• strength training

• training for activities of daily living (e.g. training for dressing, cooking or self care)

• computer-based games provided they only involve a headset, mouse or keyboard

• training in writing and the use of keyboards

• computer training (e.g. training in the use of word processors, internet or computer games)

• passive or assisted active movements (e.g. provided by therapists, family, carers or devices)

• stretches (e.g. provided by therapists, family, carers or devices)

• splinting (e.g. functional splints, resting splints, active-assist splints or hand orthoses)

• pressure garments or bandaging for oedema management or for the promotion of a passive tenodesis grip (e.g. JOBST gloves or pressure bandaging)

• arm ergometry

The use of computer games that involve hand and upper limb movement, for example games associated with Nintendo Wii^®^, PlayStation^® ^or similar equipment, will be limited where possible during the 8-week intervention period. There will be no restrictions past the 8-week intervention period.

#### Follow-up period

At the end of the 8-week intervention period, both experimental and control participants will continue to receive usual care up until the 12-month follow-up assessment. This may, or may not, involve sessions with a therapist. Therapy will not be standardised or restricted in any way. Instead, it will be left to the discretion of the treating therapists associated with the SCI units and care providers following discharge. The only restriction will be that neither experimental nor control participants use the instrumented exercise workstation. In addition, no participant will be permitted to practice any aspect of the outcome measures. They may, however, practice activities similar to those included in the hand tests as part of functional training.

#### Quality assurance

To ensure that the treatments are of a high standard and are delivered in accordance with the trial protocol, therapists responsible for administering the intensive training to experimental participants will attend a two-day workshop where they will be trained in the delivery of the treatment program. They will also be provided with a written protocol and standardised recording documents. In addition, all treatments provided to both experimental and control participants will be carefully recorded. For example, the following variables will be recorded during each treatment provided to experimental participants: therapy time, proportion of time spent with the stimulation activated, difficulty of games played and proportion of time spent playing games. Likewise usual care provided to both control and experimental participants will be recorded using a standardised form (the Spinal Cord Injury-Interventions Classification System)[11].

### Outcome assessments

Measurements will occur at baseline, 8 weeks after the commencement of the intervention period (8 weeks), and then at 6 months and 12 months after randomisation (see Table 1). There is a 3-week period after randomisation when all participants receive usual care prior to the commencement of the 8-week intervention period. This is to allow time for the delivery of the FES garments for experimental participants (these are custom made in Canada). For this reason, the 8-week assessments are 8 weeks after commencement of the intervention period but 11 weeks after randomisation.

**Table 1:** Timeline of participants' progression through the trial

week -3	week -1	week 0	weeks 0 - 3	weeks 4 - 12	week 13 ^†^	weeks 14 - 25	week 26^§^	weeks 27 - 51	week 52^§^
screening	baseline assessment by blinded assessor	concealed random allocation	usual care for all participants^€^	8-week intervention **Exp**: Instrumented exercise workstation, FES and usual care **Control**: usual care	8-week assessment by blinded assessor	usual care for all participants	6-month assessment by blinded assessor	usual care for all participants	12-month assessment by blinded assessor

^† ^plus or minus 1-week window for commencement; 1-week window for completion upon commencement

^§ ^plus or minus 1-week window for commencement; 1-week window for completion upon commencement

^€ ^this 3-week period is to allow time for the ordering and delivery of the personalised FES garment for experimental participants

All assessments will be made by research therapists blinded to group allocation. Any inadvertent unblinding of assessors will be reported. In addition, the success of blinding will be estimated by asking assessors to guess participants' group allocations at the completion of each post-randomisation assessment.

### Primary outcome

The primary outcome is the:

#### Modified Action Research Arm Test (ARAT) for the target hand at 8 weeks

The ARAT is a standardised measure of unilateral hand and upper limb function. It consists of four sub-tests including grasp, grip, pinch and gross movement. Participants will be required to perform every task in each subtest [12]. All tasks will be scored on a 4-point scale from 0 to 3 where 0 reflects poor hand function and 3 reflects good hand function. Scores will be summed to give a total possible score of 57 where a larger number reflects better hand function. The modified non-standardised table height will be used and testing will be done in a seated position. Although the ARAT was originally used as a measure of arm and hand function after stroke,[13] it has been successfully used in a trial similar to this one conducted in people with established SCI [personal communication; Prochazka A, 2009]. The ARAT has excellent reliability and has been validated against a number of other upper limb function tests [14-17]. It also has good face validity, assessing a range of functional hand tasks.

### Secondary outcomes

The secondary outcomes are the:

#### Modified Action Research Arm Test (ARAT) for the target hand at 6 months and 12 months

The ARAT will be performed and scored as described above.

#### Summed Upper Limb Strength of the target hand at 8 weeks, 6 months and 12 months

The strength component of the Graded and Redefined Assessment of Strength, Sensibility and Prehension (GRASSP) will be used to assess upper limb strength of the target hand [18]. This consists of a 6-point manual muscle test [19] to score the following nine joint actions: shoulder flexion, elbow flexion, elbow extension, wrist extension, finger flexion, finger extension, finger abduction, thumb flexion, and thumb opposition. Scores will be summed to give a total possible score of 65 where a higher score indicates better strength than a lower score. Testing will be done in a seated position.

#### AIS Sensory Assessment of the target hand at 8 weeks, 6 months and 12 months

The AIS sensory assessment is part of the assessment for the International Standard for Neurological Classification of Spinal Cord Injury. It involves testing pin-prick and light touch sensation at key points representing each cervical dermatome. Pin-prick and light-touch sensation of each dermatome is separately scored on a 3-point scale. Scores will be summed to give a total possible score of 32 where a higher score indicates better sensation than a lower score.

#### AsTex^® ^Sensory Test of the target hand at 8 weeks, 6 months and 12 months

The AsTex^® ^sensory test assesses the texture discrimination capabilities of the thumb and fingertips. It requires participants to run their thumbs, index fingers and little fingers along a grooved acrylic surface with logarithmically decreasing spaces and to stop when they perceive it as smooth [20]. The mean texture discrimination index (TDI) of the finger, thumb and little finger will be derived from three trials for each digit. The results will be interpreted with respect to age-related normative values where a lower score reflects better texture discrimination than a higher score.

#### AuSpinal Assessment of the target hand at 8 weeks, 6 months and 12 months

The AuSpinal Hand Assessment is a unilateral measure of hand function [21]. It consists of a number of hand-related tasks using the following objects: a key, nut/bolt, coin, credit card, sweet, telephone receiver and soft drink can. Scores will be summed to give a total possible score of 86 where a higher score reflects better hand function than a lower score.

#### The Goal Attainment Scale (GAS) of the target hand at 8 weeks

The GAS captures improvements on self-selected goals [22-24]. Prior to baseline assessments, participants will identify two personal goals related to use of their target hands in conjunction with their treating therapists. The goals will be set according to the SMART principle, that is, the goals will be specific, measurable, attainable, realistic and timely [25]. Participants will rate their perceptions of attainment at the 8-week assessment with the assistance of blinded assessors. The two goals will be rated on a 5-point scale, where "0" denotes the expected level of achievement; "+1" and "+2" are respectively "a little" and "a lot" better than expected, whilst "-1" and "-2" are correspondingly "a little" and "a lot" less than expected. The scores for the two goals will be averaged with a higher score reflecting better achievement of goals than a lower score.

#### The Capabilities of Upper Extremity (CUE) of the target hand at 8 weeks, 6 months and 12 months

The CUE is an interview-based questionnaire about perceptions of upper limb function specifically designed for participants with tetraplegia. Fifteen questions of the CUE related to unilateral hand and upper limb function will be used. The questions address reaching and lifting, pulling and pushing, wrist actions as well as hand and finger actions. Participants will rate their abilities to perform the 15 items on a seven-point scale from 1 to 7 [26]. Scores will be summed to give a total possible score of 105 where a higher score reflects better upper limb function than a lower score.

#### Assessment of Quality of Life - 8 (AQoL - 8) at 8 weeks, 6 months and 12 months

The AQoL-8 is a health-related quality of life instrument [27,28]. It is a self-administered 8-item questionnaire that provides utility scores varying between -0.04 (worse than death), 0 (death) and 1 (perfect health) where a higher score reflects better quality of life than a lower score.

#### Health Utilities Index Mark 3 (HUI3) at 8 weeks, 6 months and 12 months

The HUI3 is a self-administered questionnaire of health-related quality of life [29]. It covers eight attributes (vision, hearing, speech, ambulation, dexterity, emotion, cognition and pain) with five or six levels for each attribute. It is widely used in population health surveys, clinical studies and cost-utility analyses. HUI3 discriminates various aspects of burden associated with chronic conditions and describes the differences in overall health-related quality of life levels. Like the AQoL, it provides utility scores varying between -0.36 and 1 where a higher score reflects better quality of life than a lower score.

#### The self-care subscale of the Spinal Cord Independence Measure - Version III (SCIM) at 8 weeks, 6 months and 12 months

The SCIM was designed specifically for patients with SCI. The SCIM focuses on the ability to perform basic everyday tasks and takes into consideration the economic burden of disability as well as the impact of disability on overall medical condition and comfort [30]. The self-care subscale consists of six items which address ability to feed (score 0 to 3), bathe upper body (scored 1 to 3), bathe lower body (scored 1 to 3), dress upper body (scored 1 to 4), dress lower body (scored 1 to 4) and groom (scored 0 to 3). Scores will be summed to give a total possible score of 20 where a higher score reflects more independence than a lower score.

#### Participant Perception of Treatment Effectiveness at 8 weeks

Participants will be required to rate their perceptions about changes in hand function on a 15-point scale where zero indicates no change, +7 indicates "a very great deal better" and -7 indicates "a very great deal worse" [31].

#### Economic outcomes

Economic evaluation will determine whether the experimental intervention is more cost-effective than the control intervention. Cost-effectiveness analysis will measure incremental costs in the two groups in relation to the ARAT, health utility and quality of life measures. The primary focus will be the health care sector. All relevant costs associated with delivery of experimental and control interventions will be used. The cost of treatment will be estimated using standard costs for therapy and actual costs of training equipment expressed as a mean cost of treatment per participant. Community-based resource use in the 12-month follow-up period will include data on visits to GPs, specialists or other health care providers, pharmaceutical costs, as well as data on resource use specifically relating to levels of independent functioning (e.g. aids, equipment, community services, home help, home maintenance, meals on wheels, transport, formal and informal care).

#### Burden of the experimental interventions on participants

At the 8-week assessment, all participants will be asked by the blinded assessors to rate on a 10- point category rating scale their perceptions about the convenience or inconvenience of the hand training received to the target hand. This will be used to gauge the burden of the experimental intervention on participants. It will not be used as an outcome measure.

### Statistical analyses

#### Sample Size

A sample size of 78 (i.e. 39 per group) will be used based on 80% power, a between-group minimally worthwhile treatment effect on ARAT scores at 8 weeks of 5.7 points, a two-sided hypothesis test, an alpha level of 5%, a standard deviation of 14 points [personal communication; Prochazka A, 2009], an ANCOVA model that includes baseline ARAT score as a covariate, a correlation between baseline and 8 weeks ARAT scores of at least 0.8, and an adjustment to allow for a drop-out rate of 10%. All data are based on the results of a similar pilot study conducted in Canada [personal communication; Prochazka A, 2009]. Stata software (Version IC 10, StataCorp, College Station, TX) was used for sample size calculations.

#### Analysis

The primary analysis of ARAT score at 8 weeks will be performed using an ANCOVA model that includes treatment group and site as factors, and the baseline ARAT score as a covariate. All secondary outcomes (including the ARAT at 6 months and 12 months) will be analysed using a mixed model repeated-measures (MMRM) approach where applicable (i.e. where data are collected at 8 weeks, 6 months and 12 months). Multiple imputation analysis will be performed to account for the effect of missing data.

Analyses will be performed by a blinded and independent statistician according to the intention-to-treat principle and using the full dataset comprising all randomised participants. In addition, the primary analysis on the 8 weeks ARAT data will be repeated using a per protocol dataset. This dataset will only comprise participants who adhered to all aspects of the protocol and received at least 80% of training sessions (i.e., control participants who received at least 80% of the 15-minute sessions of one-to-one hand therapy and experimental participants who received this as well as 80% of the training sessions with the instrumented exercise workstation and FES). All analyses will be performed using Stata (Version 11 or higher).

### Data integrity and management

Data will be stored electronically on a database with secured and restricted access. Data transfer will be encrypted and any information capable of identifying individuals removed.

### Withdrawal

A participant will be considered to have withdrawn from the trial when consent is revoked or if the participant cannot be contacted or located. If this occurs, no further assessments will be performed. Participants will not be withdrawn from the trial for protocol violations.

### Monitoring

The trial will be overseen and monitored by a program manager. The program manager will visit each site to examine trial procedures, ensure data quality and monitor compliance with the trial protocol. Three safety variables will be monitored and documented throughout the trial. These are self-reported pain (using an 11-point category rating scale), blood pressure and skin irritation from the stimulating electrodes. However, only two safety variables (pain and blood pressure) are considered serious enough to warrant inclusion in the safety interim analysis. This analysis will be undertaken when 40 patients have completed the 8-week assessment. It will be done by an Independent Data Safety Monitoring Board comprising a statistician and two rehabilitation doctors. If there are concerns about the safety of participants, this board will make a recommendation to the trial steering committee about continuing, stopping, or modifying the trial. The Haybittle-Peto procedure for generating early stopping boundaries will be used. A recommendation of early termination due to experimental treatment inferiority on pain (mean margin of 4/10) or blood pressure (mean margin of 40 mmHg) will be considered by the Independent Data Safety Monitoring Board if the corresponding Haybittle-Peto boundary (p = 0.003, Z = 3) at a given interim analysis is crossed. No formal interim analyses for efficacy or futility are planned.

## Discussion

This trial will provide information about the effectiveness of an intensive task-specific hand training program provided with FES through an instrumented exercise workstation. Hand function in people with tetraplegia is central to their quality of life. Any treatment that can improve their hand function has the potential to make real and important differences to the lives of those affected by SCI.

This trial will adhere to key methodological principles important for minimising bias and will be reported according to the CONSORT guidelines. For example, allocation will be concealed and randomised, assessors will be blinded and analyses will be performed on an intention-to-treat basis. Therapists and participants will not be blinded due to the nature of the intervention.

One primary outcome and a number of secondary outcomes will be used. The primary outcome reflects unilateral hand function. The secondary outcomes include measures of impairment, activity limitation and participation restriction, and encompass both objective measures as well as participants' perceptions.

It is anticipated that this trial will take three years to complete. Recruitment commenced in December 2009 with the first participant randomised in February 2010. Recruitment will continue until mid 2011. The one-year follow up assessments will be completed in 2012.

## Competing interests

An Associate Investigator on the grant application has commercial interests in the instrumented exercise workstation and FES technology being used as part of this trial. MPG has a commercial interest in the AsTex^®^.

## Authors' contributions

LAH, MPG and SAD were responsible for the design of the trial and secured funding. They are also responsible for the co-ordination of the trial. LC is responsible for statistical design and analysis and YH is responsible for the cost-effectiveness analyses. All authors have read and approved the final manuscript.

## Consent

Written informed consent was obtained from the patient for publication of Figure 1. A copy of the written consent is available for review by the Editor-in-Chief of this journal.
